# Crystallographic analysis reveals the structural basis of the high-affinity binding of iophenoxic acid to human serum albumin

**DOI:** 10.1186/1472-6807-11-18

**Published:** 2011-04-18

**Authors:** Ali J Ryan, Chun-wa Chung, Stephen Curry

**Affiliations:** 1 Biophysics Section, Blackett Laboratory, Imperial College, Exhibition Road, London, SW7 2AZ, UK; 2 Biomolecular Structure, Molecular Discovery Research, GlaxoSmithKline, Stevenage SG1 2NY, UK; 3 Department of Pharmacology, University of Oxford, Mansfield Road, Oxford, OX1 3QT, UK

## Abstract

**Background:**

Iophenoxic acid is an iodinated radiocontrast agent that was withdrawn from clinical use because of its exceptionally long half-life in the body, which was due in part to its high-affinity binding to human serum albumin (HSA). It was replaced by Iopanoic acid, which has an amino rather than a hydroxyl group at position 3 on the iodinated benzyl ring and, as a result, binds to albumin with lower affinity and is excreted more rapidly from the body. To understand how iophenoxic acid binds so tightly to albumin, we wanted to examine the structural basis of its interaction with HSA.

**Results:**

We have determined the co-crystal structure of HSA in complex with iophenoxic acid at 2.75 Å resolution, revealing a total of four binding sites, two of which - in drugs sites 1 and 2 on the protein - are likely to be occupied at clinical doses. High-affinity binding of iophenoxic acid occurs at drug site 1. The structure reveals that polar and apolar groups on the compound are involved in its interactions with drug site 1. In particular, the 3-hydroxyl group makes three hydrogen bonds with the side-chains of Tyr 150 and Arg 257. The mode of binding to drug site 2 is similar except for the absence of a binding partner for the hydroxyl group on the benzyl ring of the compound.

**Conclusions:**

The HSA-iophenoxic acid structure indicates that high-affinity binding to drug site 1 is likely to be due to extensive desolvation of the compound, coupled with the ability of the binding pocket to provide a full set of salt-bridging or hydrogen bonding partners for its polar groups. Consistent with this interpretation, the structure also suggests that the lower-affinity binding of iopanoic acid arises because replacement of the 3-hydroxyl by an amino group eliminates hydrogen bonding to Arg 257. This finding underscores the importance of polar interactions in high-affinity binding to albumin.

## Background

**Figure 1:**
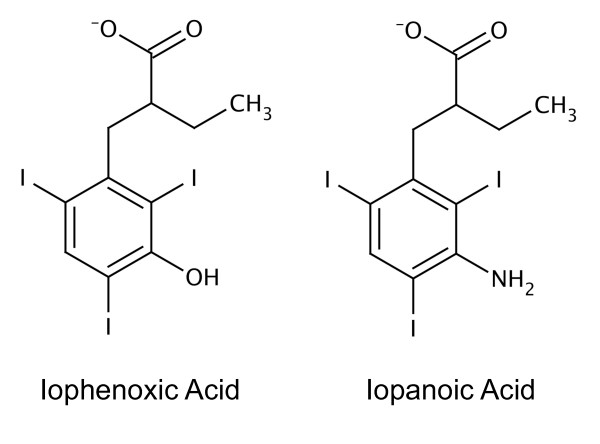
**Chemical structures of iophenoxic acid and iopanoic acid**.

Iophenoxic acid (Teridax; 2-[(3-hydroxy-2,4,6-triiodophenyl)methyl]butanoic acid) was introduced in the mid-1950s as an oral radio-contrast agent for cholecystography, a diagnostic procedure that relies on X-ray imaging of the gallbladder. This small-molecule compound, which contains three electron-dense iodine atoms, was effective for this purpose because it accumulated rapidly in the gallbladder. But iophenoxic acid was withdrawn from clinical use in 1957 after being found to have an astoundingly long half-life in the human body, estimated to be at least two and a half years [1,2]. The long residency of iophenoxic acid was thought likely to perturb both thyroid function and tests of thyroid activity that relied on measurement of the plasma concentration of the iodine-containing hormones that it secretes [3]. The compound was largely replaced by the closely related molecule, iopanoic acid. Intriguingly, although iopanoic acid only differs from iophenoxic acid by the substitution of an OH group by NH_2 _in the triiodophenyl ring (Figure 1), it has a much shorter half-life (~2 weeks) [4]. The striking difference in persistence of these two radio-contrast agents is attributed at least in part to their interactions with human serum albumin (HSA) [5].

**Figure 2:**
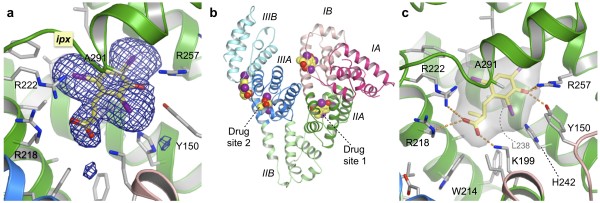
**Co-crystal structure of HSA in complex with iophenoxic acid**. (a) Simulated annealing omit map contoured at 3σ for iophenoxic acid bound in drug site 1 in sub-domain IIA. (b) Overview of HSA, coloured by sub-domain, showing the locations of the two primary drug sites and the four molecules of iophenoxic acid. (c) Close-up of iophenoxic acid bound in drug site 1. The ligand and selected side-chains are shown as sticks, coloured by atom type (carbon - grey (yellow in the iophenoxic acid); oxygen - red; nitrogen - blue; iodine - magenta). The surface of iophenoxic acid is shown as a semi-transparent grey surface. Hydrogen bonds and salt bridges are indicated by dotted orange lines.

HSA is found at very high concentrations in blood plasma and tissue fluids, typically around 0.6 mM and can have a profound effect on the distribution of many natural and artificial small-molecules [2]. The protein circulates as a monomeric polypeptide that folds into three similar helical domains (I-III), each of which is split into two sub-domains (A and B) [6] (Figure 2b). Between them these six sub-domains provide binding sites for a wide variety of endogenous ligands that are predominantly apolar molecules with anionic or electronegative features and include fatty acids, bilirubin, hemin and thyroxine [2,7]. Two of the binding pockets on the protein have been identified as the primary sites for drugs and drug-like compounds [8,9]. Crystallographic analysis has shown that these pockets -- drug sites 1 and 2 -- are located in sub-domains IIA and IIIA respectively [10,11], although additional drug binding sites have also been found [10,12-14].

Iophenoxic acid is one of the tightest binding artificial ligands of HSA (K_d _~ 20 nM) [2,4,15]. A high affinity for albumin is an important contributory factor to its persistence in blood plasma since this severely limits the free concentration of the compound, which, for reasons that are still not well understood, exerts an unusually powerful restriction on the rate of excretion of the compound [3].

Competition binding experiments showed that iophenoxic acid most readily displaces compounds that are specific for drug site 1 (*e.g*. warfarin) and strongly suggest its primary binding site is within sub-domain IIA [9,16]. The compound also competes, though less effectively, with drugs that are specific for drug site 2 (*e.g*. diazepam) [17], indicating weaker binding to this site [4]. In contrast, the structurally-related iopanoic acid (Figure 1) binds to HSA with much lower affinity (K_d _~ 150 nM) [19,20] and appears to bind preferentially to drug site 2, since it is most readily displaced by dansylsarcosine [8] and diazepam [15].

Though no longer administered to humans, the exceptional retention time of iophenoxic acid has led to it being used as a marker for tracking a variety of wild mammals including raccoons [18], badgers [19], swine [20] and Arctic foxes [21]. The compound presumably also binds to the albumins found in the plasma of these creatures. In addition, related compounds including iopanoic acid are being studied as possible treatments for hyperthyroidism [22].

We were therefore curious to understand how iophenoxic acid binds to drug site 1 on HSA with a high affinity that iopanoic acid cannot match despite its close structural similarity. To that end we attempted to determine the crystal structure of defatted HSA in complex acid with both compounds. Although HSA-iopanoic acid co-crystals failed to grow, we succeeded in determining the crystal structure of HSA-iophenoxic acid and this provides a clear explanation for the disparate binding affinities of the two iodinated compounds.

## Results and Discussion

HSA-iophenoxic acid complexes crystallised isomorphously with defatted HSA in a P1 space-group that contains two molecules in the asymmetric unit. The crystal structure was solved by molecular replacement at a resolution of 2.75 Å and strong electron density was observed for iophenoxic acid binding in drug sites 1 and 2 (Figure 2a, b). In these sites the iodine atoms could be positioned accurately by contouring the electron density map at 6σ; at 3σ density for the 2-ethyl-propanoate group became evident, allowing an unambiguous determination of the orientation of the ligand in each case.

Weaker density for the iodine atoms of iophenoxic acid was observed (at 1.5-2.5σ) in two additional binding sites, one within sub-domain IB and one in a shallow cleft between sub-domains IIIA and IIIB (Figure 2b). However, for both these sites, although the electron density for the iodine atoms clearly establishes the binding location, it does not give an indication of the other features of the ligand molecule. Consequently, these iophenoxic acid molecules have been built into the model in indicative orientations that avoid steric clashes and are consistent with the local physicochemistry.

The model was refined to yield a final R_free _value of 23.9% (Materials and Methods). Full data collection and refinement statistics are given in Table 1.

**Table 1:** Data collection and refinement statistics

**Space-group**	**P1**
**a, b, c (Å)**	55.07, 55.44, 119.85
**α, β, γ (°)**	81.00, 90.83, 64.52
**Resolution range (Å)**	59-2.75
**Independent reflections**	31655
**Multiplicity**^**1**^	1.9 (1.9)
**Completeness (%)**	95.5 (92.9)
**I/σ**__**I**__	8.2 (1.7)
**R**_**merge **_**(%)**^**2**^	9.1 (34.5)
	
**MODEL REFINEMENT**	
***Nonhydrogen atoms***	
**R**_**model **_**(%)**^**3**^	19.6
**R**_**free **_**(%)**^**4**^	23.2
**r.m.s bonds (Å)**	0.009
**r.m.s bond angles (°)**	1.18
**Average overall B-factor (Å**^**2**^**)**	46.2
**Average ligand B-factors (Å**^**2**^**)**^**5**^	18.0, 36.2, 87.1, 111.9
**Ramachandran plot (% favoured/allowed)**	89.9/8.4
**PDB ID**	2ydf

^1^Values for the outermost resolution shell are given in parentheses.

^2^*R*_*merge *_= 100 × Σ_*h*_Σ_*j*_*|I*_*hj *_*- I*_*h*_*|/*Σ_*h*_Σ_*j*_*I*_*hj *_where *I*_*h *_is the weighted mean intensity of the symmetry related reflections *I*_*hj*_

^3^*R*_*model *_= 100 × Σ_*hkl*_*|F*_*obs *_*- F*_*calc*_*|/*Σ_*hkl*_*F*_*obs *_where *F*_*obs *_and *F*_*calc *_are the observed and calculated structure factors respectively.

^4^*R*_free _is the *R*_model _calculated using a randomly selected 5% sample of reflection data omitted from the refinement.

^5^Average B-factors given for iophenoxic acid ligands bound to sub-domains IIA, IIIA, IB and in the IIIA-IIIB sites respectively.

### Drug site 1

**Figure 3:**
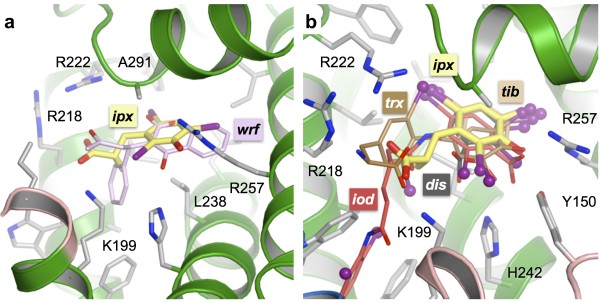
**Comparative binding of iophenoxic acid and other ligands to drug site 1 of HSA**. (a) Superposition of iophenoxic acid and warfarin [23]. Y150 is omitted from this figure to improve the visibility of the ligands. (b) Superposition of iophenoxic and other iodinated compounds that bind to drug site 1, including iodipamide (iod), tri-iodobenzoic acid (tib), di-iodosalicylic acid (dis) and thyroxine (trx) [10,24,30].

Iophenoxic acid fits snugly into the main chamber of drug site 1 where, with the exception of the carboxylate group, it is entirely shielded from solvent (Figure 2c). As observed for many other aromatic compounds [10,23], the centre of the tri-iodinated ring is contacted on either side by the side-chains of Leu 238 and Ala 291 (Figure 3a). In particular, it is worth noting that the radiocontrast agent binds is a similar position and orientation to other compounds that are composed primarily of iodinated aromatic rings, such as tri-iodobenzoic acid, di-iodosalicylic acid and iodipamide [10,24] (Figure 3b); for all three compounds there is a common locus for at least two iodine atoms (three in the case of iodipamide).

The bound conformation allows all the polar features of iophenoxic acid to make specific interactions with polar side-chains of residues lining the pocket in sub-domain IIA. The hydroxyl group on the aromatic ring makes a total of three hydrogen bonds with Tyr 150 and Arg 257; the position of these amino acids in relation to iophenoxic acid suggests the ligand hydroxyl donates a single bond to the OH group on Tyr 150 and, accepts two from the guanidinium group of Arg 257 (all 2.7-2.9 Å) (Figure 2c). At the other end of the molecule, the side-chains of Lys 199 and Arg 222 on opposite sides of the pocket entrance make salt-bridges to the carboxylate group on iophenoxic acid.

Our structure is consistent with results from competition binding experiments that identified drug site 1 as the primary high-affinity binding site for iophenoxic acid [9,16]. The near complete desolvation of the compound in the pocket and the comprehensive matching of polar groups provide a plausible account for the high affinity of iophenoxic acid for this binding site.

### Drug site 2

**Figure 4:**
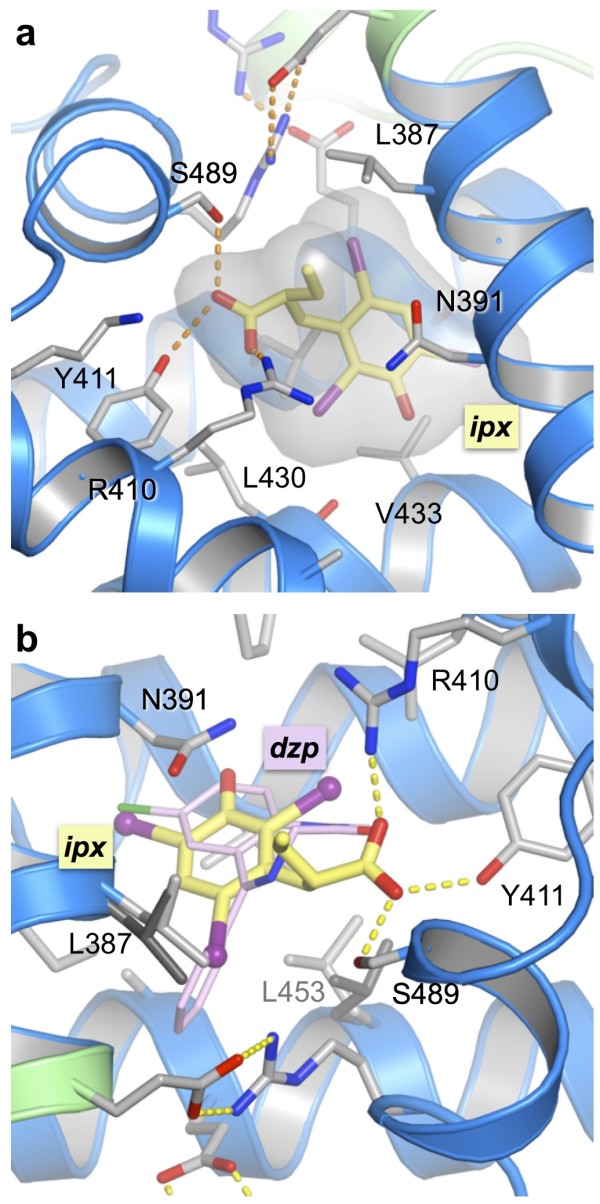
**Structural details of iophenoxic acid bound to drug site 2 in sub-domain IIIA**. (a) Close-up of iophenoxic acid bound in drug site 2, using the same representation of features as in Figure 2c). (b) Superposition of iophenoxic acid and diazepam (dzp) [10]. The side-chain conformations of L387 and L453 in the HSA-diazepam complex are shown as dark-grey sticks.

There are many similarities in the binding of iophenoxic acid to drug sites 1 and 2. In drug site 2 the compound is completely enclosed except for the partial exposure of the carboxylate at the pocket entrance, where it makes specific interactions -- though in this case they are hydrogen bonds, not salt-bridges -- with the side-chains of Tyr 411 and Ser 489 (Figure 4a). The hydroxyl group is again positioned deep within the pocket but, although it is in the vicinity of the backbone carbonyl of Leu 430 [10], it is too far away (4.4 Å) to make a hydrogen bond interaction. In contrast to the smaller propofol molecule (see Figure 2 of ref [25]), the steric hindrance caused by the three bulky iodine atoms prevents iophenoxic acid from rotating to a position that would allow the hydroxyl group to form a hydrogen bond with this group.

Two of the iodine atoms of iophenoxic acid in drug site 2 are accommodated within the main chamber, while the third inserts partially into a sub-chamber that was first observed in the structure of the HSA-diazepam structure (Figure 4b). The sub-chamber is accessed as a result of displacement of the side-chains of Leu 387 and Leu 453 by the phenyl group of diazepam [10]; however, in the case of iophenoxic acid the conformational alteration is more modest -- only the side-chain of Leu-387 has to be rotated to accommodate one of the iodine atoms of the compound (Figure 4b).

It therefore appears that a combination of factors -- absence of a hydrogen bonding partner for the hydroxyl group, substitution of hydrogen bonds for salt-bridges interacting with the carboxylate group and perhaps also the induced conformation change in Leu 387 - contribute to the lower affinity of iophenoxic acid for drug site 2 in comparison with drug site 1 [4,17].

### Other binding sites

Because of the markedly reduced strength of the electron density for the third and fourth sites, it seems likely that these are only occupied as a result of the high concentrations of iophenoxic acid used to prepare co-crystals and unlikely that they are physiologically relevant binding sites. Moreover, since the orientation of the compounds cannot be unambiguously assigned, it is difficult to provide detailed information on the mode of binding. However, the orientation of the tri-iodinated ring is clearly discerned and it is worth noting that iophenoxic acid binds to a site in sub-domain IB that was previously observed to be a secondary binding site for indomethacin, a drug that also binds primarily to drug site 1. This location overlaps with the outer part of the observed common binding site of 4*Z*, 15*E*-bilirubin-IX_α _and fusidic acid [13], an observation that may explain why iophenoxic acid can displace bilirubin from HSA [15,26].

The fourth iophenoxic molecule binds to a shallow surface cleft between sub-domains IIIA and IIIB, and partially overlaps a site that was observed to be a site of secondary binding for the drug oxyphenbutazone in the ternary HSA-myristate-oxyphenbutazone complex [10].

### The difference between Iophenoxic and Iopanoic acid binding

Although we were unable to obtain diffraction-quality co-crystals of HSA in complex with iopanoic acid, the structure of the HSA-iophenoxic acid complex provides a plausible explanation for the difference in binding behavior of these very similar iodinated compounds, particularly with regard to drug site 1. Iophenoxic acid binds with high affinity (K_d _~ 20 nM) to this site because it is a good fit to the dimensions of the pocket and makes specific interactions with its polar groups (Figure 2c). In particular its hydroxyl group can form three hydrogen bonds with Tyr 150 and Arg 257 deep within the pocket. The replacement of this hydroxyl group with an NH_2 _group in iopanoic acid is a very modest structural change, so the compound is likely to bind in the same orientation as observed for iophenoxic acid. This assumption is supported by drug site 1 modeling experiments performed with the docking software, GOLD, which found that seven of the highest-scoring solutions docked iopanoic acid in the pocket in an orientation very similar to iophenoxic acid and placed the NH_2 _group close to the side-chains of Tyr 150 and Arg 257. However, the NH_2 _group in iopanoic acid has less hydrogen binding capability in this position than the hydroxyl group in iophenoxic acid. The low pK_a _value of an NH_2 _substituent on an aromatic ring means that it is likely only to be able to donate hydrogen bonds, so the compound would only be able to make a single hydrogen bond, with Tyr 150.

The observed mode of interaction of iophenoxic acid with drug site 1 on HSA is reminiscent of a similar finding for CMPF (3-carboxy-4-methyl-5-propyl-2-furanpropanoic acid), a more polar compound which nevertheless also binds to this site with good affinity (K_d _= 100 nM) [27] as a result of specific interactions involving all the polar groups on the ligand [10]. Thus it appears that ligands with significant polar features can bind to HSA with high affinity if their hydrophilic groups can all find specific interacting partners within the binding site.

As well as reducing its binding affinity for site 1, this structural difference in iopanoic acid also appears to confer a preference for drug site 2 in sub-domain IIIA, though its selectivity for this binding pocket is less marked than the site 1/site 2 selectivity of iophenoxic acid [28]. Given the close structural similarity between the two compounds, it seems likely that iopanoic acid could bind to drug site 2 in precisely the same orientation as observed for iophenoxic acid. In this case, the NH_2 _group would still be too far from the carbonyl group of Leu 430 (or any other nearby carbonyl) to make a hydrogen bond. While this may account for its lower affinity (K_D _~ 150 nM) for the protein [4], it remains a puzzle why iopanoic acid preferentially binds to drug site 2, especially since the NH_2 _group would be predicted to hydrogen bond to Tyr 150 in drug site 1. The factors governing the differential binding of this compound to drug sites 1 and 2 on albumin are evidently quite complex.

## Conclusions

The crystal structure of the HSA-iophenoxic acid complex reveals a total of four binding sites on the protein, only two of which are likely to be physiologically relevant. It indicates that high-affinity binding to drug site 1 is likely to be due to extensive desolvation of the compound, coupled with the ability of the binding pocket to provide a full set of salt-bridging or hydrogen bonding partners for its polar groups.

Recognition of the hydroxyl group on the iodinated benzyl ring by the side-chains of Tyr 150 and Arg 257 appears to be particularly crucial to binding. The replacement of this hydroxyl by an amino group in iopanoic acid is predicted to lower the binding affinity by reducing the number of hydrogen bonds made with Tyr 150 and Arg 257.

## Methods

Recombinant HSA was kindly donated by Prof. Eishun Tsuchida (Waseda University, Japan) and was defatted and purified in preparation for crystallisation as described previously [10]. Iophenoxic acid was obtained from Sigma Aldrich at the highest available purity and was solubilised in dimethylsulphoxide (DMSO). HSA-iophenoxic acid complexes were prepared using established methods [10]: HSA at 100 mg/ml in 50 mM potassium phosphate, pH7 was mixed with sufficient 100 mM iophenoxic acid in DMSO to achieve a 5:1 molar ratio of the compound to the protein. This mixture was incubated with rotation at room temperature for an hour and the excess, unbound iophenoxic acid was removed by repeated cycles of dilution with 50 mM potassium phosphate, pH7 containing 20 μM iophenoxic acid in a 10 kD MW cut-off centrifugal ultra-filtration device. The complex was crystallised at 100 mg/ml from 24-30% (w/v) polyethylene glycol 3350 as reported [10,25].

X-ray diffraction data were collected at room temperature from capillary-mounted crystals on beamline 14.1 at SRS Daresbury, using methods established in our laboratory for HSA crystals [7]. Data processing and scaling were performed using MOSFLM and SCALA from the CCP4 suite [29]. The crystals grew isomorphously to the P1 crystals previously obtained for defatted HSA [10,25,30] and the data were phased by molecular replacement using a previously determined high-resolution structure of defatted HSA (PDB 2bxa) stripped of its ligands [10]. Rounds of model building in O [31] were interleaved with cycles of refinement with CNS [32]. The structure of iophenoxic acid used in model building was obtained from the Cambridge Structural Database via the Chemical Database Service [33]. Final rounds of refinement, which included translation, libration and screw-rotation (TLS) refinement using groups defined by the TLS Motion Determination web-server [34] were performed with Phenix [35].

For docking experiments, structures of iophenoxic acid and iopanoic acid were generated in ChemBio3D 12.0 (CambridgeSoft, 2009) the structures then underwent energy minimisation in Avogadro http://avogadro.openmolecules.net/. Docking was carried out into the structure of HSA (PDB: 2BXA[10]) stripped of all of its ligands. Docking of iophenoxic acid and iopanoic acid into HSA was carried out using GOLD 5.0.1 [36]. Docking solutions were obtained for positions within a radius of 20 Å of the hydroxyl of Tyr 150. All amino acid side chains were set as rigid during the docking while the ligand was allowed to be flexible.

## Abbreviations

HSA: Human Serum Albumin; DMSO: dimethylsulphoxide;

## Authors' contributions

AJR performed the structure determination and drafted the manuscript; CC contributed to experimental design and revision of the manuscript; SC contributed to experimental design, crystallographic refinement and interpretation and revised the draft manuscript. All authors have read and approved the final manuscript.
